# How Do Prebiotics Affect Human Intestinal Bacteria?—Assessment of Bacterial Growth with Inulin and XOS In Vitro

**DOI:** 10.3390/ijms241612796

**Published:** 2023-08-14

**Authors:** Nelly Schropp, Virginie Stanislas, Karin B. Michels, Kerstin Thriene

**Affiliations:** Institute for Prevention and Cancer Epidemiology, Faculty of Medicine and Medical Center, University of Freiburg, 79110 Freiburg, Germany; nelly.schropp@uniklinik-freiburg.de (N.S.); virginie.stanislas@uniklinik-freiburg.de (V.S.); karin.michels@uniklinik-freiburg.de (K.B.M.)

**Keywords:** prebiotics, inulin, XOS, xylooligosaccharides, microbiota, growth curve, growth modeling, complex carbon source, commensal bacteria, saccharolytic fermentation

## Abstract

Prebiotics are believed to exhibit high specificity in stimulating the growth or activity of a limited number of commensal microorganisms, thereby conferring health benefits to the host. However, the mechanism of action of prebiotics depends on multiple factors, including the composition of an individual’s gut microbiota, and is therefore difficult to predict. It is known that different bacteria can utilize inulin and xylooligosaccharides (XOS), but an overview of which bacteria in the human gut may be affected is lacking. Detailed knowledge of how bacterial growth is affected by prebiotics is furthermore useful for the development of new synbiotics, which combine a living microorganism with a selective substrate to confer a health benefit to the host. Hence, we developed a statistical model to compare growth in vitro among typical human gut bacteria from different phylogenetic lineages. Based on continuous observation of the optical density (OD_600_), we compare maximal growth rates (r_max_), maximal attained OD_600_ (OD_max_), and area under the growth curve (AUC) of bacteria grown on inulin or XOS. The consideration of these three parameters suggests strain-specific preferences for inulin or XOS and reveals previously unknown preferences such as *Streptococcus salivarius* growth on XOS.

## 1. Introduction

In recent years, the human gut microbiota and its role in human health has gained increasing attention. The modulation of the gut microbial composition through diet has been the focus of numerous studies [1,2,3]. In particular, prebiotics appear to have a profound impact on the gut microbiota and human health by acting as “substrates that are selectively utilized by host microorganisms conferring a health benefit” [4]. These dietary compounds resist digestion by the host and stimulate the growth and/or activity of certain commensal microorganisms. Non-digestible polysaccharides are currently the best-studied prebiotics [5]. Among them, inulin and XOS (xylooligosaccharides) are well-known, commercially available representatives. Inulin is a prebiotic fiber that occurs naturally in many plants such as chicory, agave, jerusalem artichoke, onions, garlic, bananas, wheat, and asparagus. The linear molecule inulin consists of fructose units that are usually bound to a terminal glucose molecule [6]. XOS consist of xylose units and occur naturally in bamboo shoots, fruits, vegetables, milk, and honey [7]. Compared to inulin, one of the best-known prebiotics that is already widely used in the food industry, XOS are rather emerging prebiotics with the advantage of lower dose requirements to be used [8,9]. This has been hypothesized to be caused by fewer bacteria being able to ferment XOS [9]. The metabolization of prebiotic oligo- or polysaccharides by the gut microbiota is referred to as saccharolytic fermentation and leads mainly to the formation of short-chain fatty acids (SCFAs), which are associated with health benefits for the human host [10]. Inulin has been reported to stabilize blood sugar, reduce blood lipids and inflammation, increase mineral absorption, contribute to weight loss, relieve constipation and depression, and even prevent colon cancer [8]. Similarly, XOS has been attributed with anti-inflammatory, anti-tumor, antioxidant, and anti-microbial effects [11].

Although the composition of human gut microbiota differs greatly between individuals, Firmicutes (also known as Bacillota), Bacteroidetes (Bacteroidota), Actinobacteria (Actinomycetota), Proteobacteria (Pseudomonadota), Fusobacteria (Fusobacteriota), and Verrucomicrobia (Verrucomicrobiota) are the most common phyla, with Firmicutes and Bacteroidetes accounting for about 90% of the phyla detected in adults [12,13]. The genus *Bifidobacterium*, which belongs to the phylum Actinobacteria, has been reported most commonly to increase upon inulin and XOS consumption, but results from in vivo studies vary [7,14]. A number of representatives of the Firmicutes and Bacteroidetes are also capable of utilizing these prebiotics, as previously demonstrated by in vitro experiments [15,16,17,18].

In order to effectively apply prebiotics as a potential treatment or prevention method against disease, it is essential to understand the capability of the bacteria of the human gut microbiota to utilize these prebiotics. Therefore, targeted in vitro experiments are an important approach to better understand the preferences of gut bacteria in the utilization of different prebiotics and to investigate how prebiotic administration affects bacteria that have not been previously associated with prebiotic utilization. Most in vitro studies analyzing the growth performance of individual bacterial species on inulin and XOS have focused on *Lactobacillus* (now subdivided into novel genera [19]) and *Bifidobacterium* [9,18,20] or other bacteria associated with the production of health-promoting SCFAs [15,16,21]. Some studies examining the effects of prebiotics on bacterial growth have reported inconsistent results. For example, in 2014, Scott et al. described that the growth of *B. infantis* DSM 20088 is stimulated by XOS [15], whereas in 2020, Zeybek et al. stated that *B. infantis* NRLL B-41661 does not grow on XOS in single culture [17].

The aim of this study was to investigate the ability of common human gut bacteria to utilize inulin and XOS, including species not previously associated with this feature. Therefore, we established an in vitro approach based on continuous monitoring of the optical density (OD_600_) to observe the growth of a bacterial liquid culture. We compared the area under the growth curve (AUC) on a medium supplemented with prebiotics considering the maximal growth rate (r_max_) and maximal population size (OD_max_). The use of these three parameters allowed for a detailed tracking of bacterial growth, revealing individual reactions and potential preferences for either prebiotic in our study. To our knowledge, this is the first in vitro study to incorporate all three of these parameters to investigate the prebiotic effect of inulin and XOS on human gut bacteria. With this method, we report for the first time that the *Streptococcus salivarius* strain DSM 20067 displays enhanced growth in the presence of the prebiotic XOS.

## 2. Results

**Figure 1:**
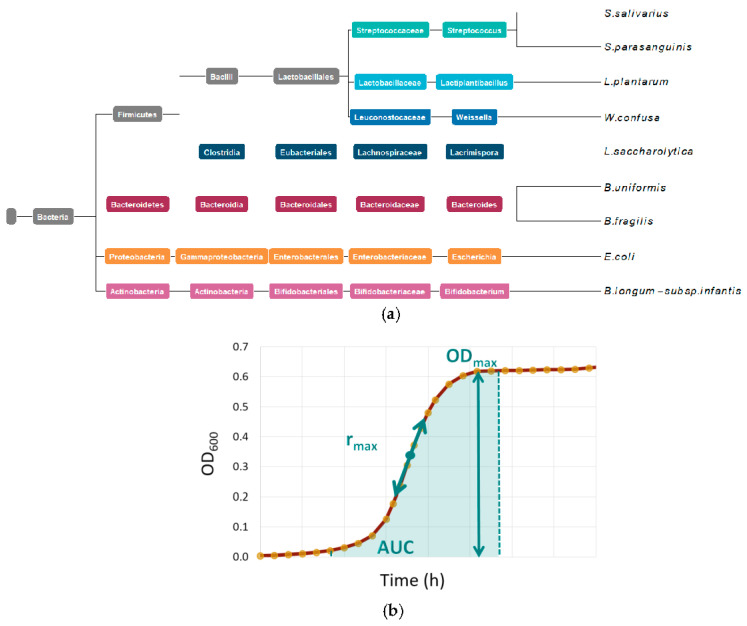
Overview of study content: (**a**) phylogenetic background of the bacterial species used in the study; colors indicate different species identity. (**b**) Calculated parameters from measured growth curves: r_max_ = maximal growth rate, OD_max_ = maximal optical density at a wavelength of 600 nm (OD_600_), AUC = area under the curve in exponential phase.

To investigate the utilization of prebiotics by human intestinal bacteria, we established an in vitro analysis based on continuous monitoring of the optical density (OD_600_) of a bacterial liquid culture in combination with a statistical approach including pairwise comparisons of the different conditions. We selected bacteria with different phylogenetic backgrounds but originating from the most common phyla in the human gut (Figure 1a). In particular, we aimed to include species that had not yet been studied in detail under these conditions and that were growing well in our laboratory. We defined three parameters (area under the growth curve (AUC), maximal growth rate (r_max_), and maximal population size (OD_max_)) derived from the obtained growth curves to perform pairwise comparisons of the growth of these bacteria under the influence of prebiotics (Figure 1b).

**Figure 2:**
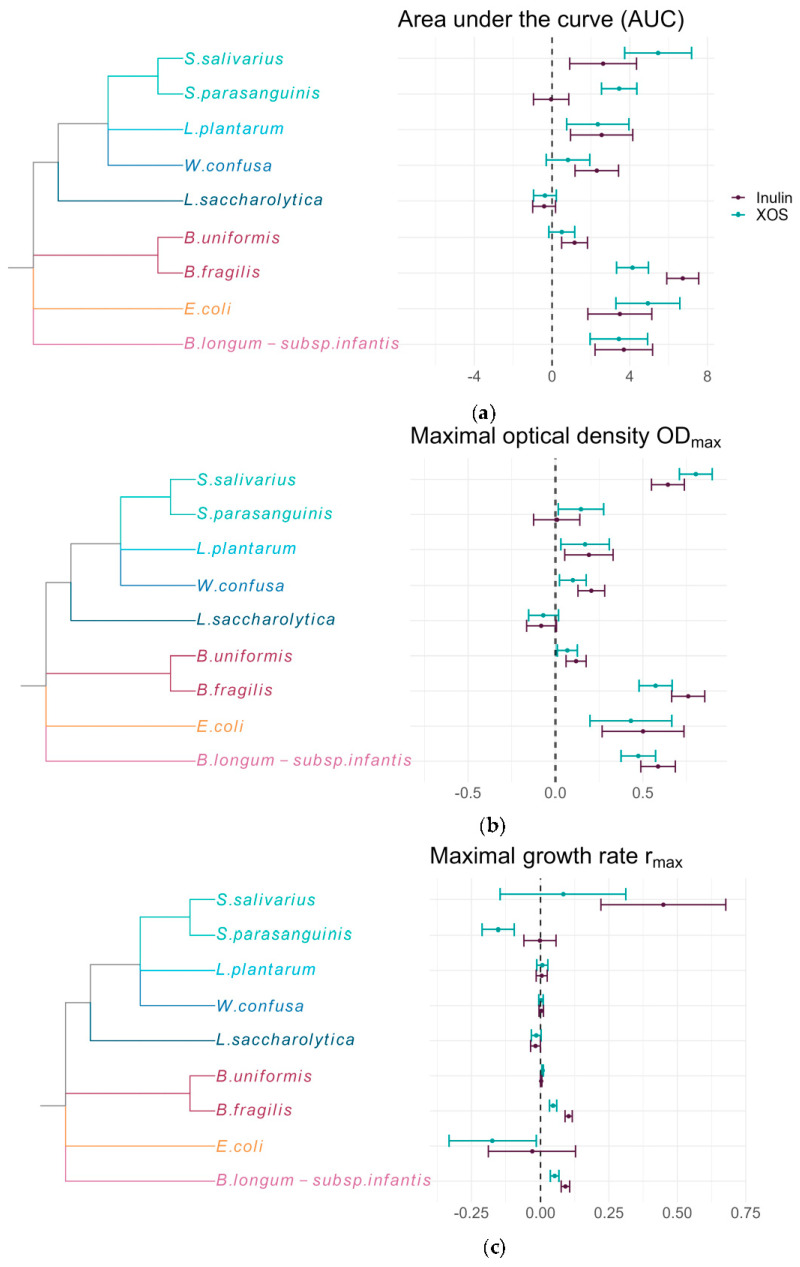
Pairwise difference in marginal means for each growth parameter and species. Depicted are the pairwise differences between each prebiotic (XOS or inulin) and control (Δc) with 95% confidence intervals: (**a**) Δc of area under the curve (AUC); (**b**) Δc of maximal reached OD_600_ (OD_max_); and (**c**) Δc of maximal observed growth rate (r_max_).

The pairwise differences in the calculated growth parameters between growth on prebiotics (XOS or inulin) and the control without added carbohydrates (Δc) are depicted in Figure 2. *S. salivarius* presented enhanced AUC, OD_max_, and r_max_ values for both prebiotics, with all Δc’s significantly increased (post-hoc tukey test < 0.05) with the exception of r_max_ values for XOS. In contrast, the growth of *S. parasanguinis* did not appear to be affected by inulin, whereas this strain grew significantly slower on XOS (as indicated by a decreased r_max_ value) and reached significantly higher OD_max_ and AUC values. *L. plantarum* displayed significantly increased AUC and OD_max_ in the presence of both prebiotics, but no significant change in r_max_. Likewise, we did not detect significant changes in r_max_ in the case of *W. confusa* when incubated with either prebiotic. However, this strain grew to significantly higher OD_max_ on either prebiotic, and the AUC was significantly increased on inulin. The parameters calculated from the growth curves of *L. saccharolytica* (formerly “*Clostridium saccharolyticum*” [22]) were all decreased, but only the Δc of the r_max_ on inulin was significant. The analysis of the growth curves of *B. uniformis* and *B. fragilis* during exposure to either prebiotic revealed an increase in all parameters compared to the control, even though for *B. uniformis*, the change in AUC values with XOS and the r_max_ with inulin were not significant. In this study, *E. coli* grew significantly slower in the presence of XOS, whereas the change in r_max_ on inulin was not significant. Despite that, AUC and OD_max_ were significantly enhanced by incubation with inulin and XOS. Both growth curves of *B. infantis* incubated with inulin and XOS exhibited a significant increase in all parameters.

To highlight the differences in growth parameters when bacteria were exposed to either prebiotic, we calculated the pairwise difference of inulin compared to XOS (Δi-x) (Table 1). Positive Δi-x values indicate greater values on inulin compared to XOS (displayed in cyan) and vice versa. The resulting values of *L. plantarum*, *L. saccharolytica*, and *E. coli* did not differ significantly when comparing the two prebiotics. In the case of *B. fragilis*, the difference in all observed parameters was significantly positive (shown in purple). *B. uniformis* grew significantly faster on XOS compared to inulin, but the overall increase in r_max_ was very low. The other two Δi-x values obtained for this species were not significant. Upon exposure to inulin, *W. confusa* exhibited significantly higher AUC and OD_max_ values. *B. infantis* enhanced OD_max_ and r_max_ values when grown on inulin. The two *Streptococcus* species reached significantly higher r_max_ when growing on inulin, but the AUC and OD_max_ resulted in the reverse.

## 3. Discussion

In this study, we present a detailed in vitro analysis investigating how the prebiotics inulin and XOS affect the growth of bacteria representing four different phyla commonly found in the human gut. The consideration of r_max_, OD_max_, and AUC in this study allowed for a detailed analysis of how inulin and XOS affect bacterial growth. Thereby, our data indicate very individual bacterial growth behavior on each prebiotic, with bacteria from different genera reacting to inulin and XOS. To our knowledge, this is the first study using a clearly defined comparison structure that considers three different growth parameters to investigate the change in growth induced by inulin and XOS. Furthermore, comparing these three parameters on the same prebiotic highlights the difficulty in defining “good growth” based on a single value.

Species that have previously been correlated with growth on XOS and inulin in vitro are *Bifidobacterium infantis, Bacteroides fragilis,* and *Lactiplantibacillus plantarum,* and this is mainly in agreement with our results [15,16,18,23,24,25]. Even though we did not observe any significant difference in the growth of *L. plantarum* with either prebiotic, our data suggest inulin to be the preferred prebiotic of *B. fragilis* and *B. infantis*, although this was less pronounced for *B. infantis*, as here, the Δi-x value of the AUC was not significant. Interestingly, Scott et al. observed higher maximal OD_650_ for *B. infantis* (DSM 20088) growing on HP inulin (consisting of molecules with a higher degree of depolymerization, i.e., a longer chain length) but not on inulin produced from dahlia compared to XOS [15]. Furthermore, *B. infantis* NRLL B-41661 was reported to not grow on XOS by Zeybek et al. [17]. Li et al. observed different strains of *B. infantis* isolated from human donors to grow on agar plates supplemented with different concentrations of XOS [18]. Thus, strain identity as well as the source and/or the processing of prebiotics are important considerations for the comparison of results from different growth experiments.

Comparing two representatives each of the genera *Bacteroides* and *Streptococcus*, we infer that the growth of strains of more closely related species differs as much as that of more distantly related strains. Although *B. uniformis* strains possess the largest repertoire of carbohydrate-active enzymes (CAZY genes) among *Bacteroides*, this strain exhibited more difficulties with growth on prebiotics than *B. fragilis*. Slow growth on inulin has been reported for a related *B. uniformis* strain in another study [26]. In contrast to the unchanged r_max_ values when growing on inulin, we observed slightly but significantly increased r_max_ values of *B. uniformis* on XOS. Even though we further observed significantly increased OD_max_ values on both inulin and XOS, plant-based polysaccharides may not be the preferred energy source of *B. uniformis* that has been positively associated with breast-feeding and mucus degradation [26]. Both Bacteroides species are considered potential probiotics [27]. Probiotics are defined as “live microorganisms that, when administered in adequate amounts, confer a health benefit on the host” [28]. However, in contrast to *B. uniformis*, *B. fragilis* not uncommonly turn pathogenic [29,30]. This highlights the challenge of a clear assignment of health promoting effects to certain bacterial species. Furthermore, the variation of strains in vivo may change rapidly depending on mutation rates, generation time, horizontal gene transfer, selection pressure, or host diet [31]. It is very difficult to predict which strains may be stimulated in vivo or to estimate how representative a particular strain is compared to the total set of all strains in the human microbiome pool belonging to a certain bacterial species.

Another emerging focus of research is the concept of synbiotics, including the administration of “mixture[s] comprising live microorganisms and substrate(s) selectively utilized by host microorganisms that confers a health benefit on the host” [32]. *Streptococcus salivarius* has been ascribed a positive role on the stability of the microbiota, but may in rare cases cause invasive opportunistic infection [33]. In a phase II clinical trial, *S. salivarius* was administered along with inulin to improve the symptoms of oral halitosis [34], but to our knowledge, there have not been any studies investigating the combination of *S. salivarius* with XOS. We report for the first time that the growth of *S. salivarius* is increased (based on OD_max_ and AUC values) when grown on XOS compared to inulin. Hence, these results suggest that XOS may be a valuable alternative for developing new synbiotics in combination with *S. salivarius*. Accordingly, we suggest further studies to investigate these promising results.

In contrast, the second representative of the genus *Streptococcus*, *S. parasanguinis,* did not display any significant change in growth on inulin in our study. In alignment with this, Whiley et al. suggested the inability of *S. parasanguinis* to utilize inulin as a substrate [35]. Interestingly, in our study, XOS significantly reduced the growth rate of *S. parasanguinis* while increasing OD_max_ and AUC.

*L. saccharolytica* was the only strain in this study that did not show any significant growth change on either prebiotic. In alignment with this, Murray et al. suggested that members of this species depend on other bacteria that are able to metabolize complex polysaccharides like inulin into sugars, which can then be utilized by *L. saccharolytica* [36]. To our knowledge, there is no literature concerning the investigation of the growth of *L. saccharolytica* on XOS.

Former studies suggest that the growth of the fermented food borne species *W. confusa* on XOS seems to be strain specific as well [37]. In our experiments, *W. confusa* (DSM 20196) was rather weakly yet significantly stimulated by XOS and inulin, but displayed a tendency to grow better on inulin compared to XOS. In alignment with this, the strain NRRL-B- 14171 has been reported to also be able to utilize inulin to some degree [38].

*E. coli* is a very ubiquitous species [39] and has been proposed to hardly be able to grow on inulin and XOS in vitro by determination of growth rate values exclusively [23,24,38,40,41]. These findings are in alignment with the r_max_ values we observed in our study. Additionally, we determined OD_max_, which increased significantly after incubation with either prebiotic. This example highlights the importance of considering all three parameters when studying bacterial growth. In general, the optical density at a wavelength of 600 mn represents the amount of living and dead cells present in the bacterial culture. Higher maximal OD_600_ (OD_max_) values thus indicate more bacterial biomass. The maximal growth rate (r_max_), on the other hand, describes how fast a prebiotic can be utilized by a bacterial species. We suggest including both parameters, r_max_ as well as OD_max_, into the investigation of bacterial growth, as well as investigating the combination of both parameters, the AUC. Our AUC values were calculated during the exponential growth phase, when the culture was most actively replicating. Both parameters influence the AUC value in opposite ways: although a higher growth rate reduces the AUC value, higher OD_max_ values increase it.

Our pairwise comparison approach provides insights into relative changes in the bacterial population attributable to the added prebiotics, but results should be classified with caution. Our results compare the capabilities of different bacteria to use inulin and XOS for growth, differentiating the prebiotic influence on growth rate and maximal optical density. In general, our study reveals that more strains that were able to grow on either prebiotic preferred inulin, suggesting that XOS might be more selective compared to inulin. This is consistent with other studies that have observed that comparatively few strains grow on XOS [15,17]. In addition, the microbial fermentation of XOS is associated with lower gas production than that of inulin, suggesting a reduced fermentability by the gut microbiota [42]. We highlight that the growth of specific bacteria may be stimulated in different ways, depending on their substrate uptake and utilization mechanisms used. For example, there are “polysaccharide generalists” (many from the genus *Bacteroides*) with the capacity to utilize a variety of polysaccharides simultaneously, and more specialized bacteria that switch to specific chain length molecules after their preferred substrate is depleted (like members of *Bifidobacterium*) [16,43]. Thus, bacterial growth in microbial communities likely differs from behaviour in single cultures.

Naturally, prebiotics occur in a variety of plants consisting of an inhomogeneous mixture of mono- and polysaccharides in varying ratios. In our study, we used commercially available prebiotics that were isolated from agave (inulin) and corn crop (XOS). They, therefore, also contained monosaccharides that might cause a different growth pattern if compared to growth on highly purified prebiotics [41]. To address this, we added a control containing 1% glucose instead of a prebiotic (5% final concentration) in each experiment to evaluate the proportion of observed growth attributable to monosaccharide consumption alone (Figures S1 and S2).

In our study, *B. fragilis* grew significantly better on inulin compared to the control containing glucose. In contrast, we could not detect stronger growth on XOS. *E. coli* grew significantly faster on glucose than on either of the prebiotics, but the resulting OD_max_ was comparable to the growth on the prebiotic substrates, whereas the AUC was significantly decreased on glucose. *S. parasanguinis* grew equally well on glucose and inulin but to significantly higher OD_max_ on XOS. The AUC further indicates stimulation by XOS even compared to glucose. It should, however, be kept in mind that this significantly enhanced AUC resulted from the slow growth on XOS. This decrease of r_max_ on XOS was, however, not significant when compared to glucose. In the case of *S. salivarius*, r_max_ on glucose lay in between the respective values on inulin and XOS, not significantly differing from each of them. The OD_max_ and AUC values, on the other hand, were significantly lower on glucose. For all other strains, we observed better or equal growth on glucose compared to growth on the two prebiotics.

In this study, we developed a statistical approach to investigate the in vitro utilization of the two prebiotics inulin and XOS by typical human gut bacteria. The limitations include the difficulty to define the representability of a bacterial strain in vivo, the neglect of bacterial interactions, and the likely presence of contaminating sugars in the used prebiotics. In summary, we detected that more strains that grew on both prebiotics preferred inulin, suggesting that XOS is more selective compared to inulin. Inulin, on the other hand, appeared to have a more profound impact on the growth rate of the human gut bacteria investigated in this study. However, the *Streptococcus* species tended to prefer XOS as a substrate. Particularly, we report here for the first time that the *Streptococcus salivarius* strain DSM 20067 displays enhanced growth in the presence of the prebiotic XOS. To our knowledge, this has not yet been demonstrated in in vivo studies. Further studies are warranted to investigate their ability to ferment XOS and evaluate potential applications of *Streptococcus* species in combination with XOS as a potential synbiotic.

## 4. Materials and Methods

### 4.1. Cultivation of Bacteria

Bacteria were purchased from the German Collection of Microorganisms and Cell Cultures (DSMZ) or kindly provided by Annette Wittmer, Institute for Microbiology and Hygiene, Faculty of Medicine and Medical Center, University of Freiburg. Strain denotations are indicated in Table 1. Cultures were grown at 37 °C for at least 24 h in an anaerobic jar containing GENbox anaer for anaerobic conditions (bioMérieux Deutschland GmbH, Nürtingen, Germany). Culture medium contained the following components: 2.5 g peptone pancreatic from casein (Merck KGaA, Darmstadt, Germany), 2.5 g trypticase peptone (Thermo Fisher Scientific GmbH, Dreieich, Germany), 5 g yeast extract (Difco 0127-17-9), 2 mL resazurin solution 0.1% (SERVA Electrophoresis GmbH, Heidelberg, Germany), 0.25 g CaCl_2_ · 2H_2_O (Merck KGaA, Darmstadt, Germany), 0.2 g MgSO4 (Merck KGaA, Germany, Darmstadt), 1.0 g K_2_HPO_4_ (Merck KGaA, Darmstadt, Germany), 1.0 g, KH_2_PO_4_ (Merck KGaA, Germany, Darmstadt), 10.0 g NaHCO_3_ (Merck KGaA, Darmstadt, Germany), 50 g NaCl (Merck KGaA, Darmstadt, Germany), 2.5 mL hemin solution 0.1% (SERVA Electrophoresis GmbH, Germany, Heidelberg), 0.05 mL vitamin K1 1% (Merck KGaA, Darmstadt, Germany), 0.25 g cysteine-hydrochloride (SERVA Electrophoresis GmbH, Germany, Heidelberg), 5 g glucose (Merck KGaA, Darmstadt, Germany), 500 mL aqua dest [44]. Low oxygen levels were monitored with the aid of Microbiology Anaerotest^®^ (VWR International GmbH, Bruchsaal, Germany) displaying low oxygen levels through discoloration of the indicator.

### 4.2. Analysis of the Utilization of Prebiotics by Bacteria

To determine the utilization of prebiotics, glucose in the growth medium was replaced by inulin (Aleavedis Naturprodukte GmbH, Bexbach, Germany, produced from agave) or XOS (Nutrasumma, Phoenix, AZ, USA, produced from corn crop) at a final concentration of 5% and a pH of 7. Prebiotics were dissolved in maximum recovery diluent (MRD; Oxoid, Basingstoke, Hampshire) and sterilized by filtration using filters with a pore size of 0.22 µm (Carl Roth GmbH + Co, Karlsruhe, Germany).

After a minimum of 24 h of pre-incubation at 37 °C under anaerobic conditions, the spent medium was exchanged for medium containing either inulin, XOS, or no carbohydrate source, respectively. For the measurement, 200 µL of each inoculated medium was transferred into a 96-well plate in triplicate. The 96-well plate was prepped with Microbiology Anearocult^®^ P (VWR International GmbH, Bruchsaal, Germany) and sealed with an adhesive tape to decrease oxygen levels inside the plate. Optical density was measured continuously at a wavelength of 600 nm (OD_600_) every 20 min starting at OD_600_ < 0.2 for at least 15 h using a spark microplate reader (Tecan, Männedorf, Swizerland). Each experiment was conducted three times, and invalid experiments were rerun to ensure that three experiments were available for each bacterial strain.

### 4.3. Statistical Analysis

All statistical analyses were performed in R (version 4.1.1). Bacterial growth was evaluated over three experiments, during which three replicates on each growth condition were measured. Three blank replicates (medium controls) were averaged as growth references for each medium in each experiment. The growth curves of each analyzed bacterial species were subsequently adjusted by withdrawing the corresponding average medium OD_600_ at each time point as well as the minimum value. The corrected growth curves were fitted to a standard form of the logistic equation using the R package growthcurver [45]. We used three parameters to describe the growth of each curve in detail (depicted in Figure 1b). The OD_max_ was directly derived from the model. Additionally, we computed the following two metrics: the maximum growth rate (r_max_) and the area under the fitted logistic curve (AUC) during the exponential growth phase. We defined the maximum growth rate as the maximum slope of the curve, that is, the maximum point reached by the derivative of the estimated growth curve. We restricted the computation of AUC to the exponential growth phase in order to compare multiple growth curves over equivalent intervals. The growth phase was defined as the time interval where the derivative of the estimated growth curve was greater than a given constant (here set to 0.005). The AUC was subsequently computed as the integral of the logistic curve over this defined interval. For each strain, the association between the type of medium and each metric was evaluated using a one-way blocked ANOVA model considering the different experiments as a blocking factor. ANOVA assumptions and residual distributions were verified for all models. We estimated marginal means for each medium and conducted pairwise comparisons using Tukey tests (R package lsmeans [45]).

## Figures and Tables

**Table 1:** Pairwise difference between inulin and XOS (Δi-x) of area under the curve (AUC), maximal OD_600_ (OD_max_), and maximal growth rate (r_max_) with 95% confidence interval [CI] for each species. Significant differences (post-hoc tukey test < 0.05) are highlighted in color; positive values are shaded in purple, negative values in cyan.

Species (Strain)	AUC [CI]	OD_max_ [CI]	r_max_ [CI]
*Streptococcus salivarius* (DSM 20067)	−2.830 [−4.551; 1.110]	−0.160 [−0.255; −0.066]	0.366 [0.137; 0.594]
*Streptococcus parasanguinis* (DSM 6778)	−3.503 [−4.413; −2.594]	−0.137 [−0.268; −0.007]	0.150 [0.091; 0.209]
*Lactiplantibacillus plantarum* (DSM 20174)	0.198 [−1.403; 1.800]	0.022 [−0.117; 0.161]	−0.002 [−0.022; 0.018]
*Weissella confusa* (DSM 20196)	1.483 [0.363; 2.600]	0.106 [0.029–0.183]	0.001 [−0.007; 0.009]
*Lacrimispora saccharolytica* (DSM 2544)	−0.042 [−0.631; 0.547]	−0.012 [−0.096; 0.072]	−0.003 [−0.021; 0.015]
*Bacteroides uniformis* (DSM 6597)	0.657 [−0.007; 1.320]	0.050 [−0.007; 0.108]	−0.005 [−0.008; −0.003]
*Bacteroides fragilis* (DSM 2151)	2.590 [1.760; 3.410]	0.188 [0.093; 0.283]	0.057 [0.043; 0.070]
*Escherichia coli* (ATCC 25922)	−1.440 [−3.090; 0.204]	0.070 [−0.165; 0.305]	0.143 [−0.015; 0.302]
*Bifidobacterium longum subsp. infantis* (DSM 20090)	0.254 [−1.180; 1.690]	0.114 [0.015; 0.213]	0.039 [0.024; 0.055]

## Data Availability

The data presented in this study are available on request from the corresponding author.

## Supplementary Materials

The following supporting information can be downloaded at: https://www.mdpi.com/article/10.3390/ijms241612796/s1.
