# Supplementary figures and images for: How Do Prebiotics Affect Human Intestinal Bacteria?—Assessment of Bacterial Growth with Inulin and XOS In Vitro
Source: Int J Mol Sci. 2023 Aug 14;24(16):12796. doi: 10.3390/ijms241612796 (PMC10454692; doi:10.3390/ijms241612796)

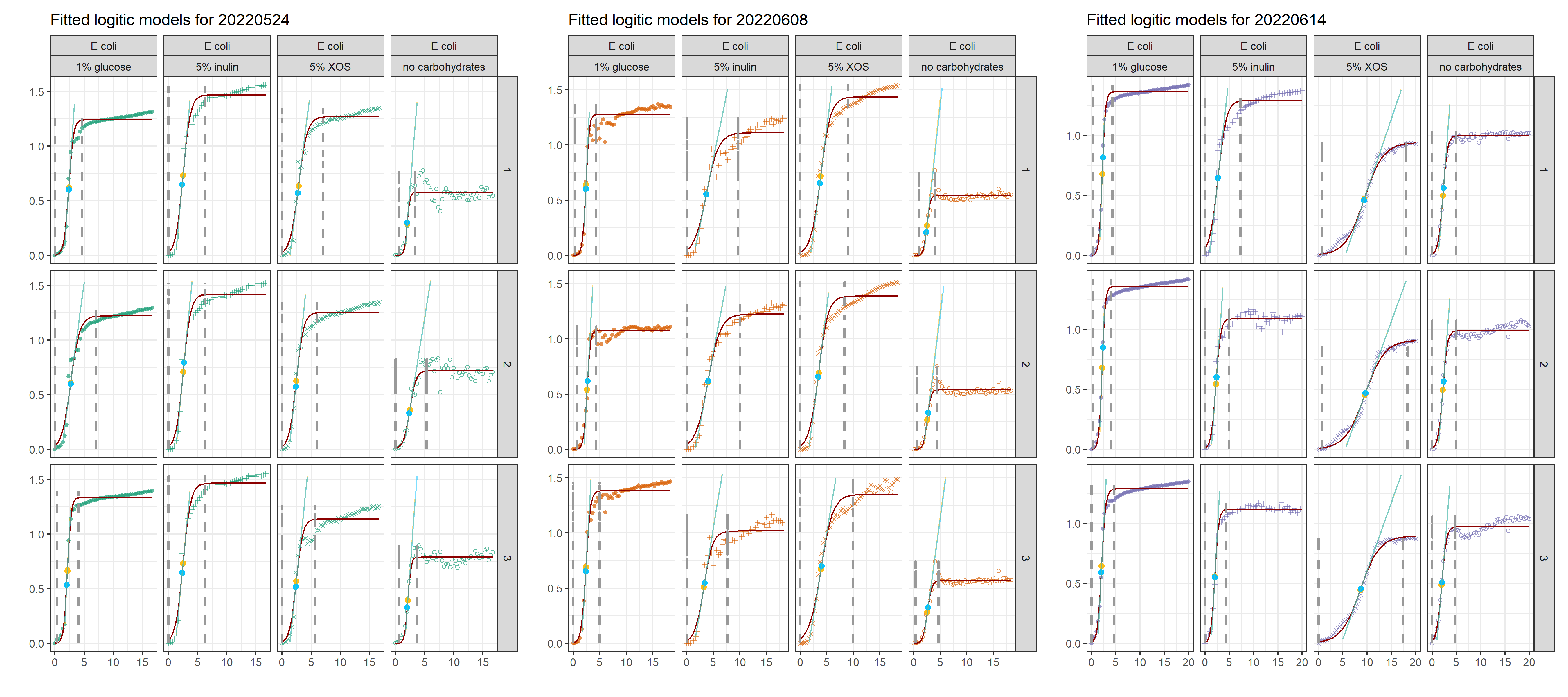

Supplement: Supplementary file 1 [file ijms-24-12796-s001.zip › supplementary-figureS1_coli_curves.png]

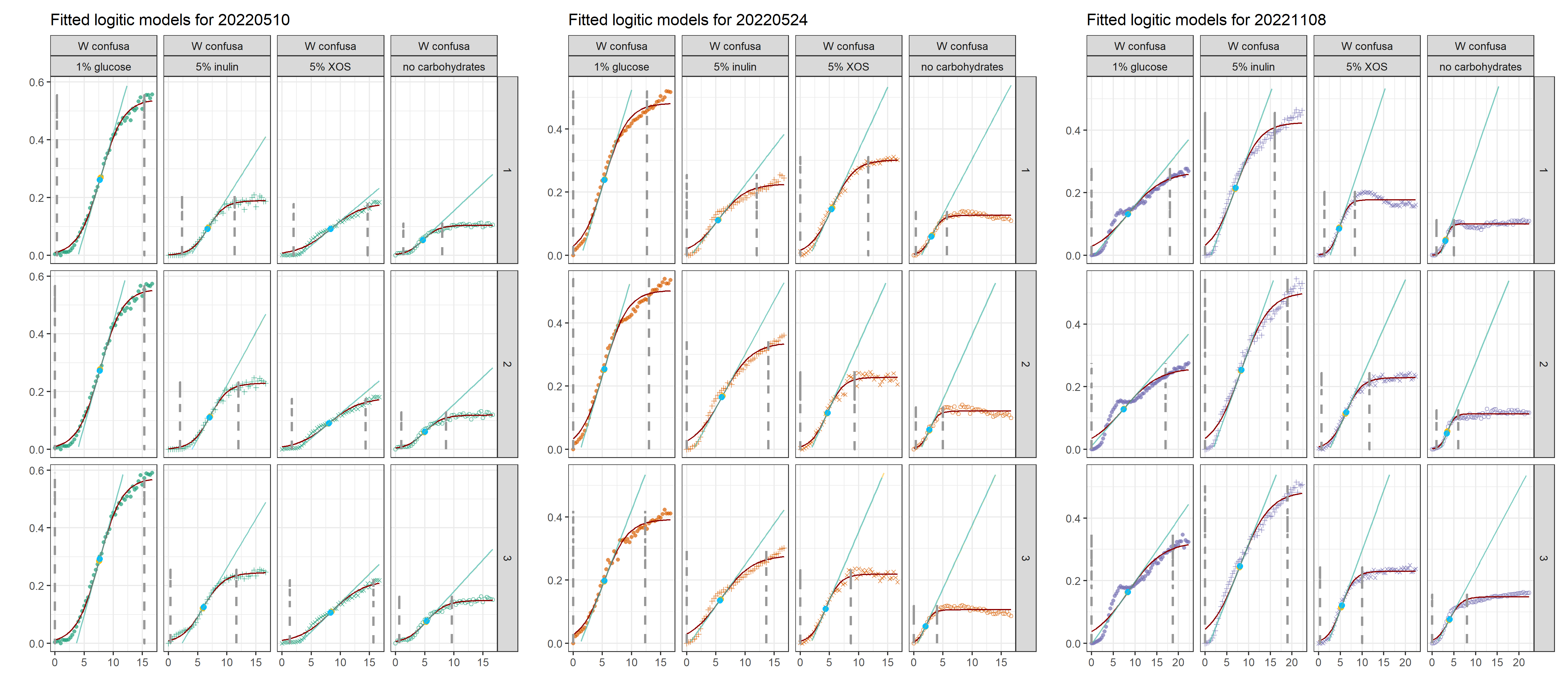

Supplement: Supplementary file 1 [file ijms-24-12796-s001.zip › supplementary-figureS1_confusa_curves.png]

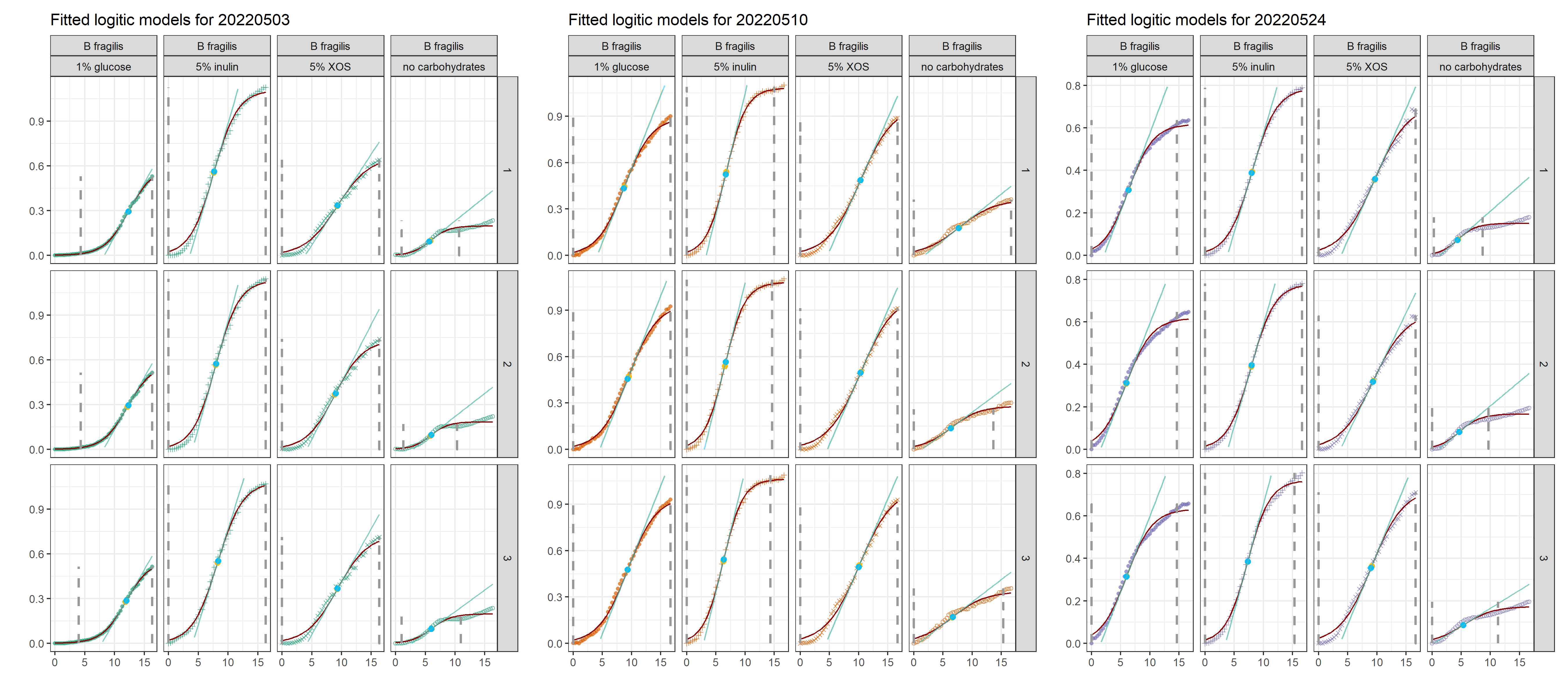

Supplement: Supplementary file 1 [file ijms-24-12796-s001.zip › supplementary-figureS1_fragilis_curves.png]

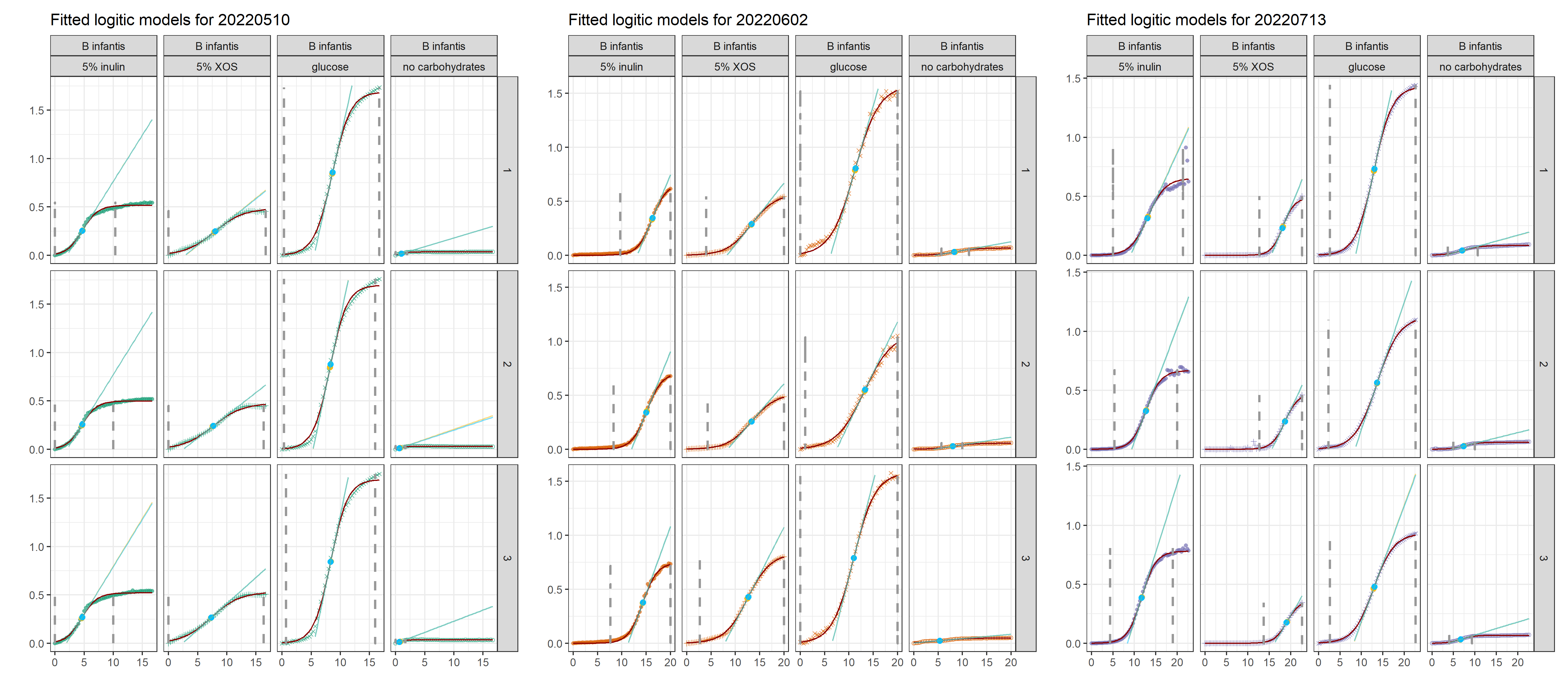

Supplement: Supplementary file 1 [file ijms-24-12796-s001.zip › supplementary-figureS1_infantis_curves.png]

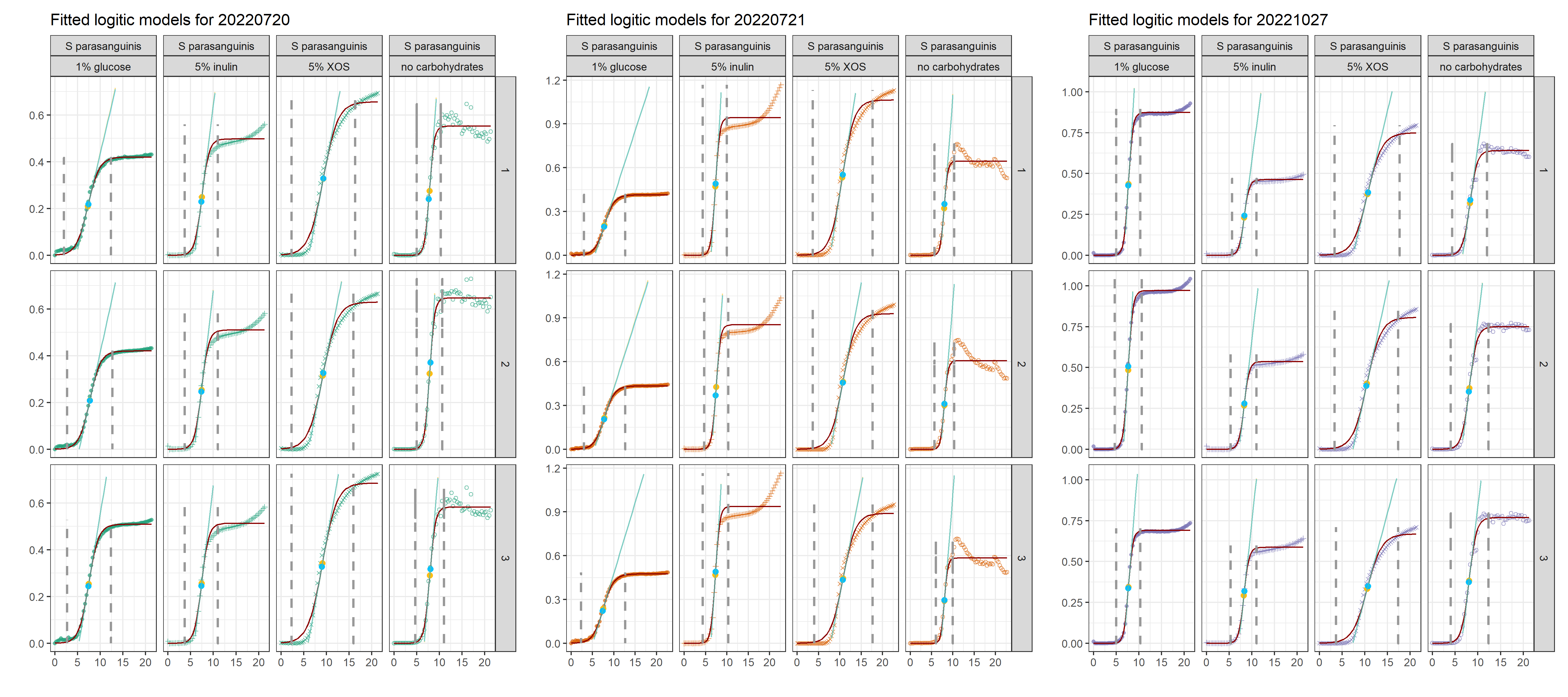

Supplement: Supplementary file 1 [file ijms-24-12796-s001.zip › supplementary-figureS1_parasanguinis_curves.png]

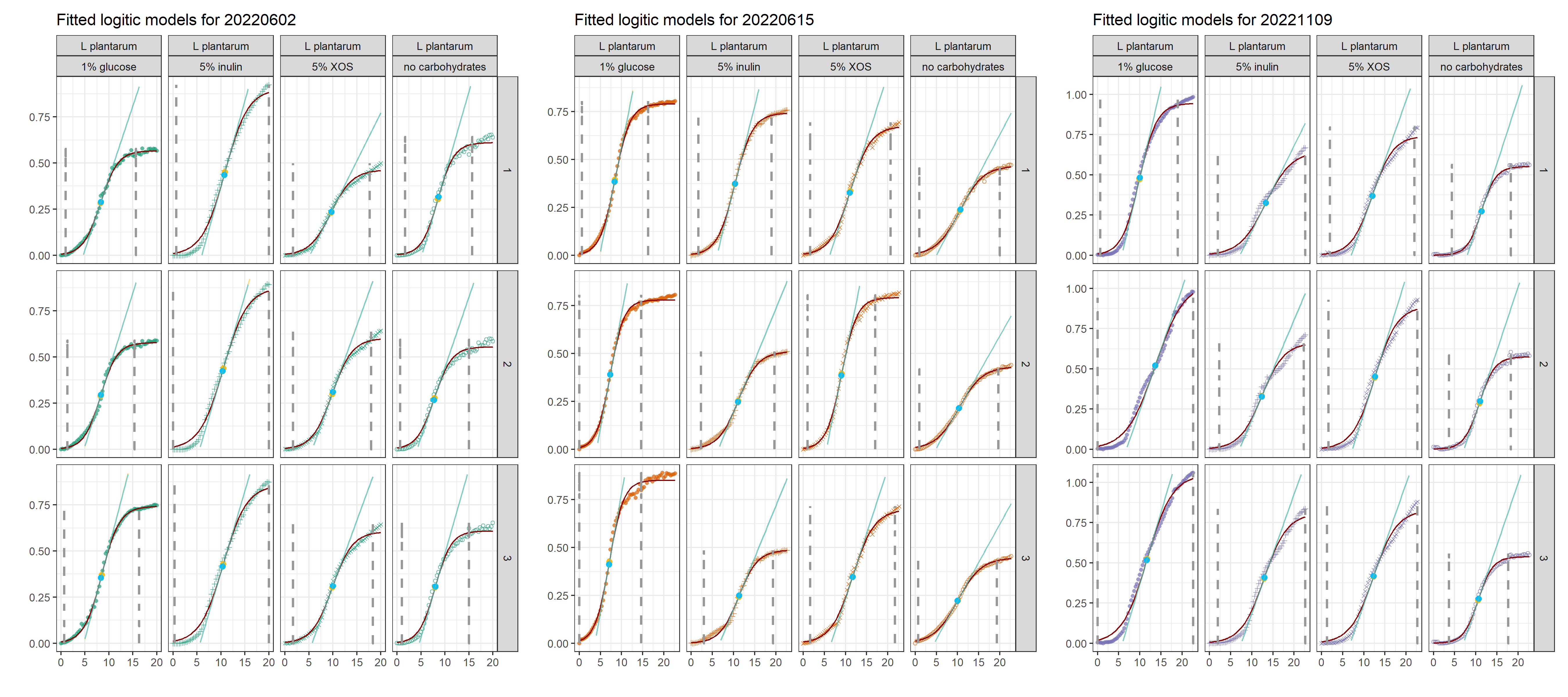

Supplement: Supplementary file 1 [file ijms-24-12796-s001.zip › supplementary-figureS1_plantarum_curves.png]

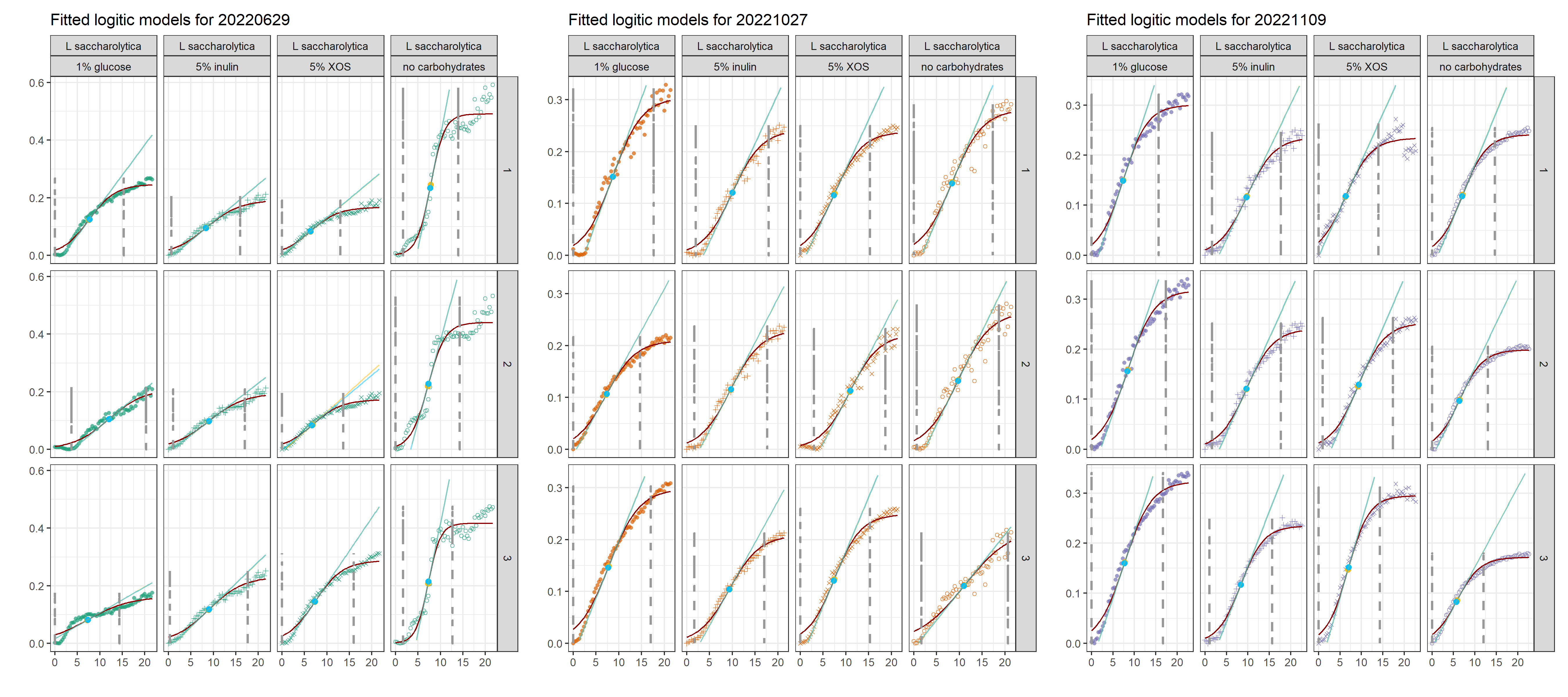

Supplement: Supplementary file 1 [file ijms-24-12796-s001.zip › supplementary-figureS1_saccharolytica_curves.png]

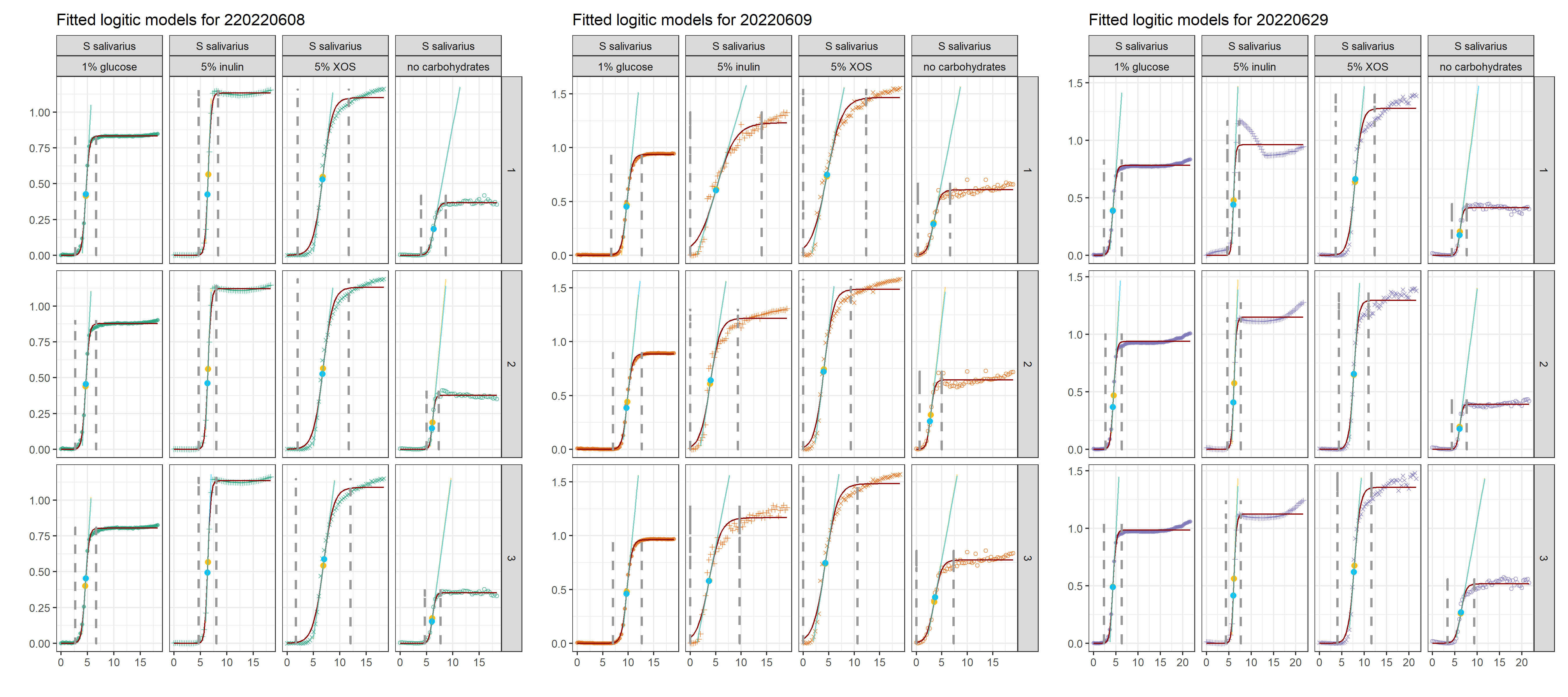

Supplement: Supplementary file 1 [file ijms-24-12796-s001.zip › supplementary-figureS1_salivarius_curves.png]

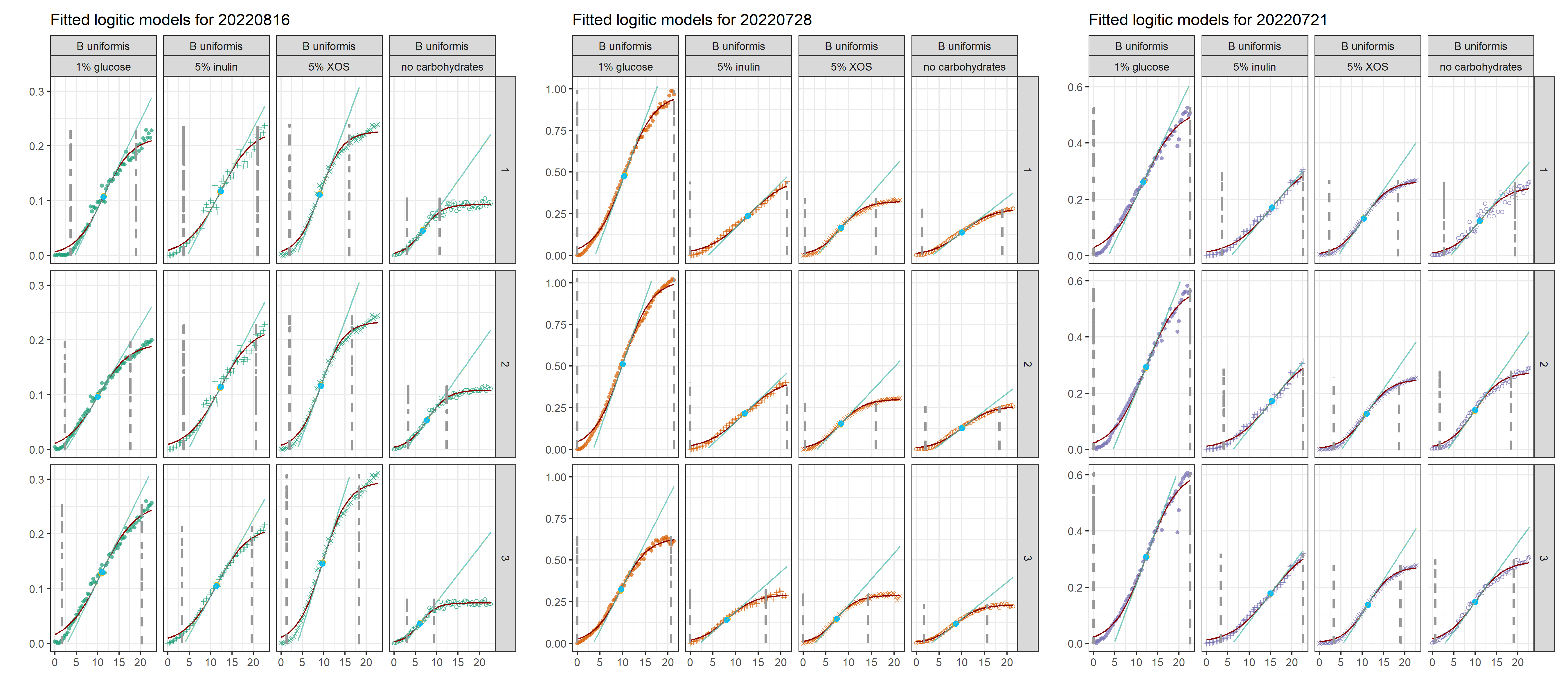

Supplement: Supplementary file 1 [file ijms-24-12796-s001.zip › supplementary-figureS1_uniformis_curves.png]

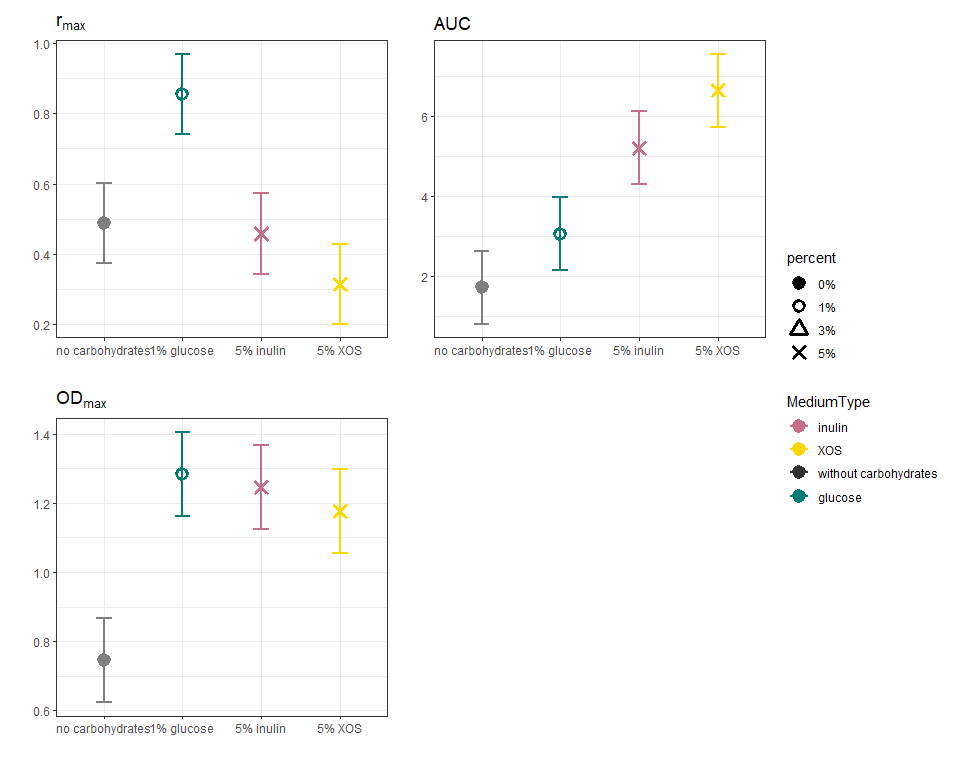

Supplement: Supplementary file 1 [file ijms-24-12796-s001.zip › supplementary-figureS2_coli_boxplots.png]

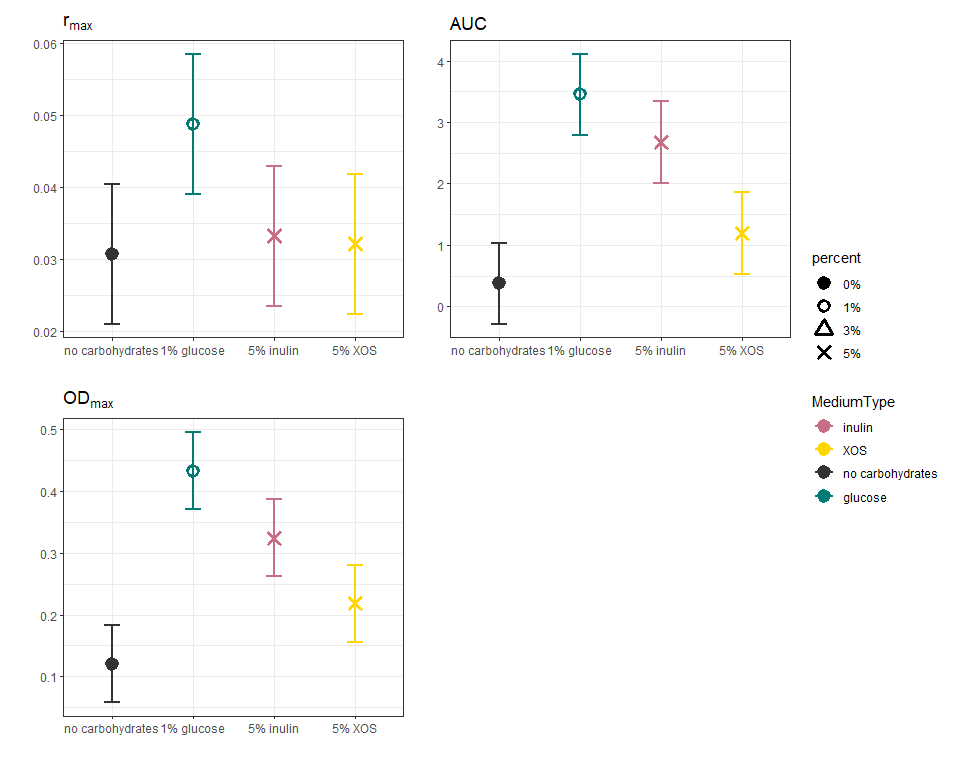

Supplement: Supplementary file 1 [file ijms-24-12796-s001.zip › supplementary-figureS2_confusa_boxplots.png]

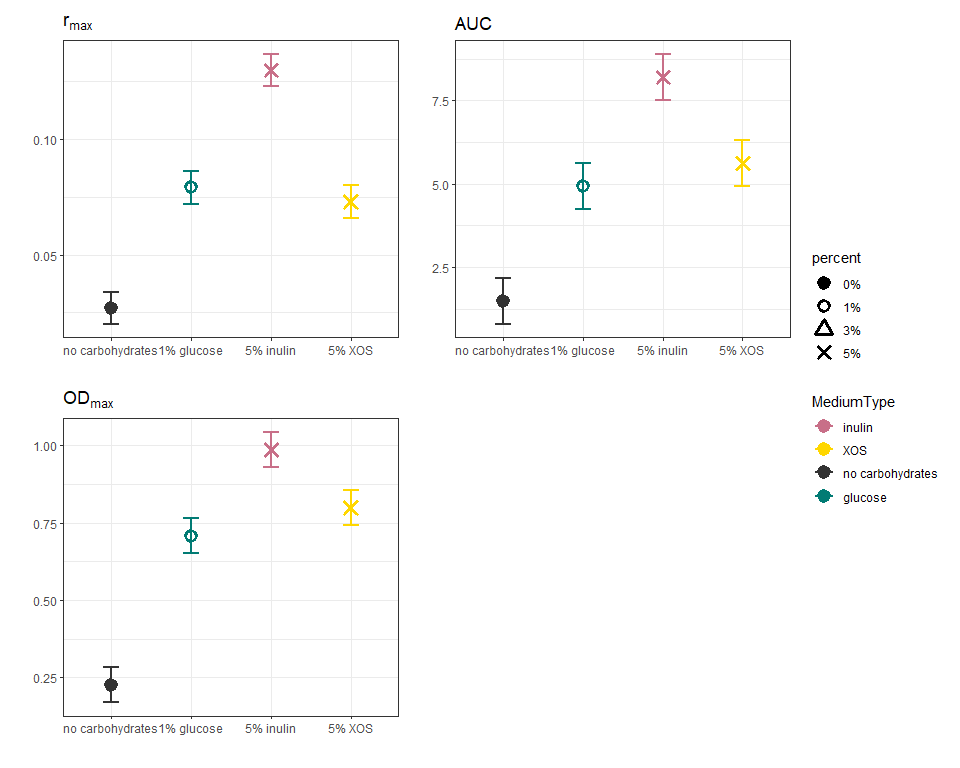

Supplement: Supplementary file 1 [file ijms-24-12796-s001.zip › supplementary-figureS2_fragilis_boxplots.png]

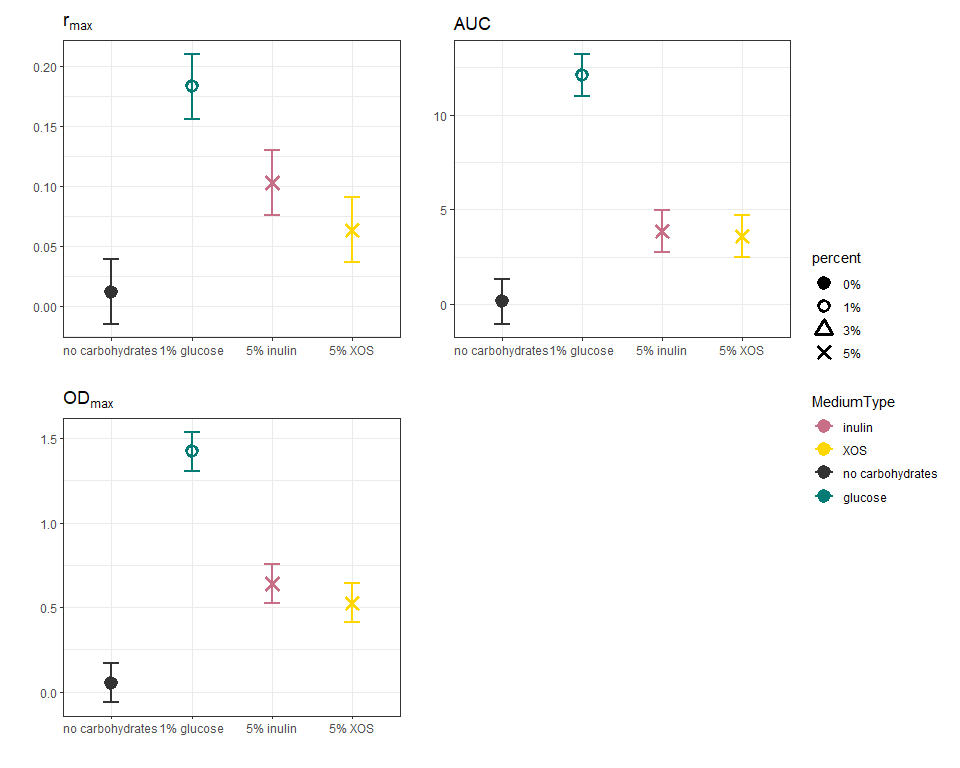

Supplement: Supplementary file 1 [file ijms-24-12796-s001.zip › supplementary-figureS2_infantis_boxplots.png]

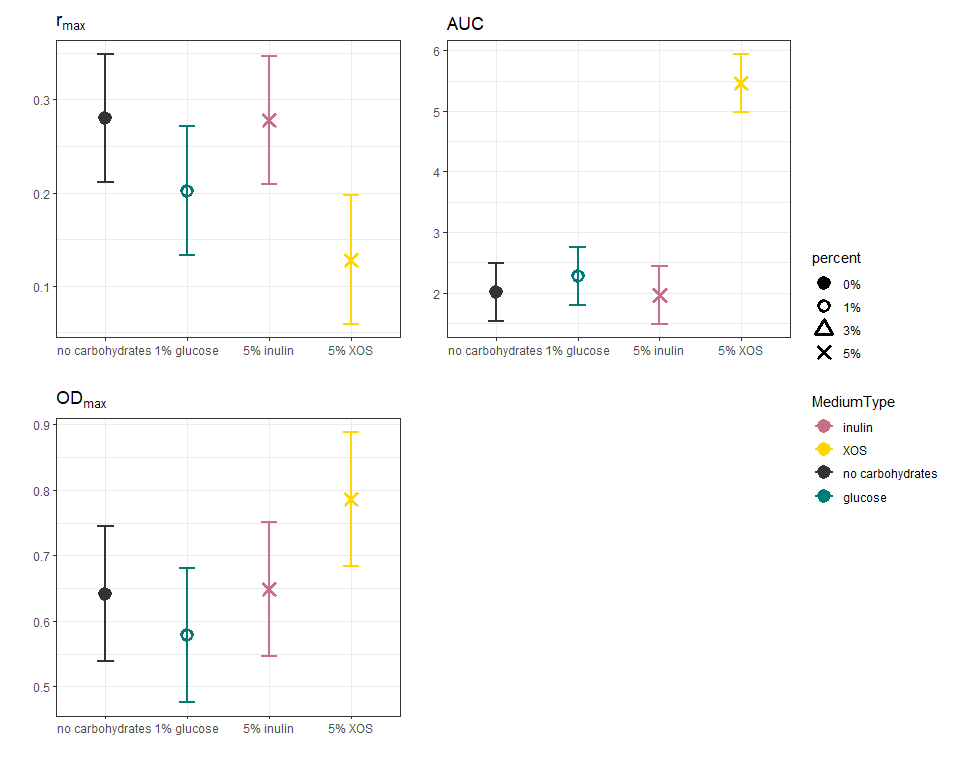

Supplement: Supplementary file 1 [file ijms-24-12796-s001.zip › supplementary-figureS2_parasanguinis_boxplots.png]

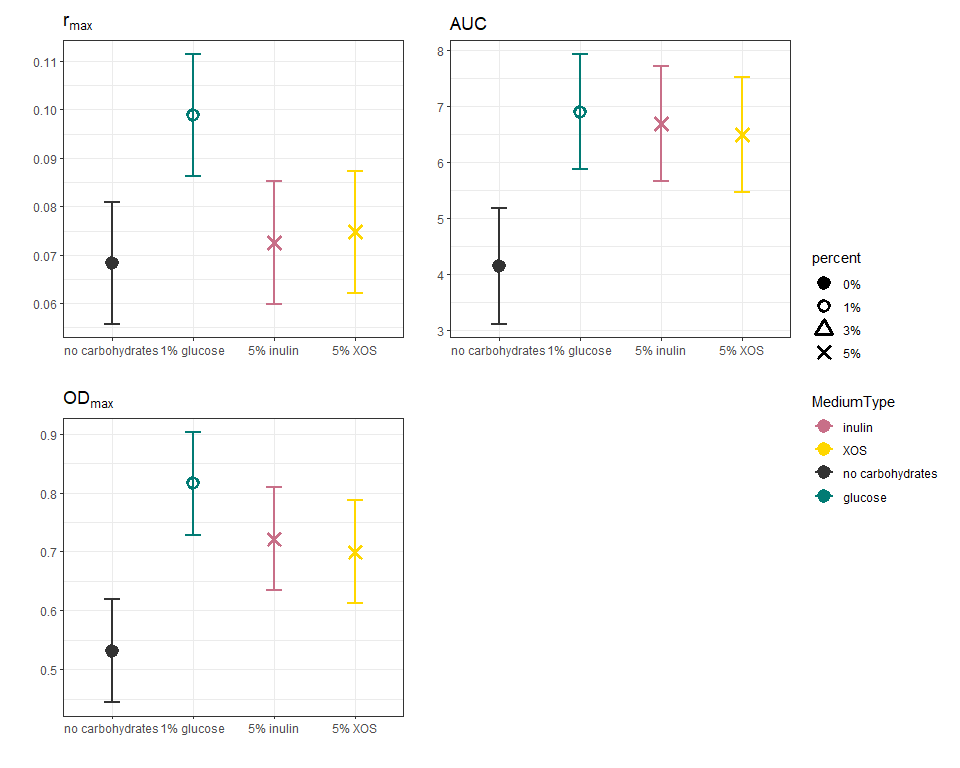

Supplement: Supplementary file 1 [file ijms-24-12796-s001.zip › supplementary-figureS2_plantarum_boxplots.png]

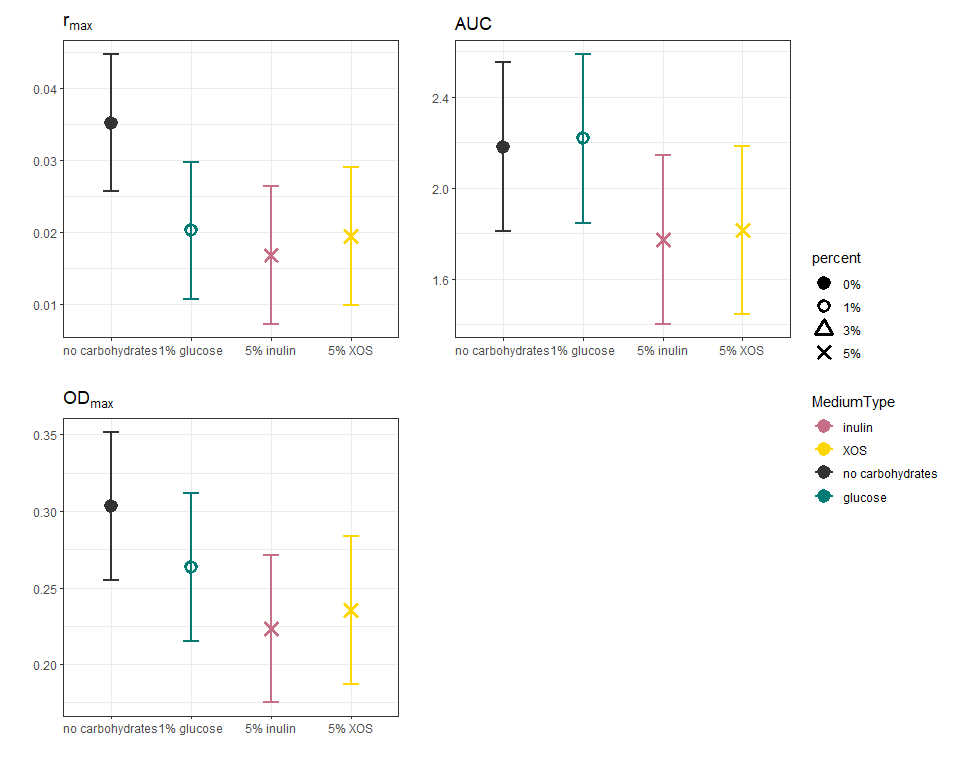

Supplement: Supplementary file 1 [file ijms-24-12796-s001.zip › supplementary-figureS2_saccharolytica_boxplots.png]

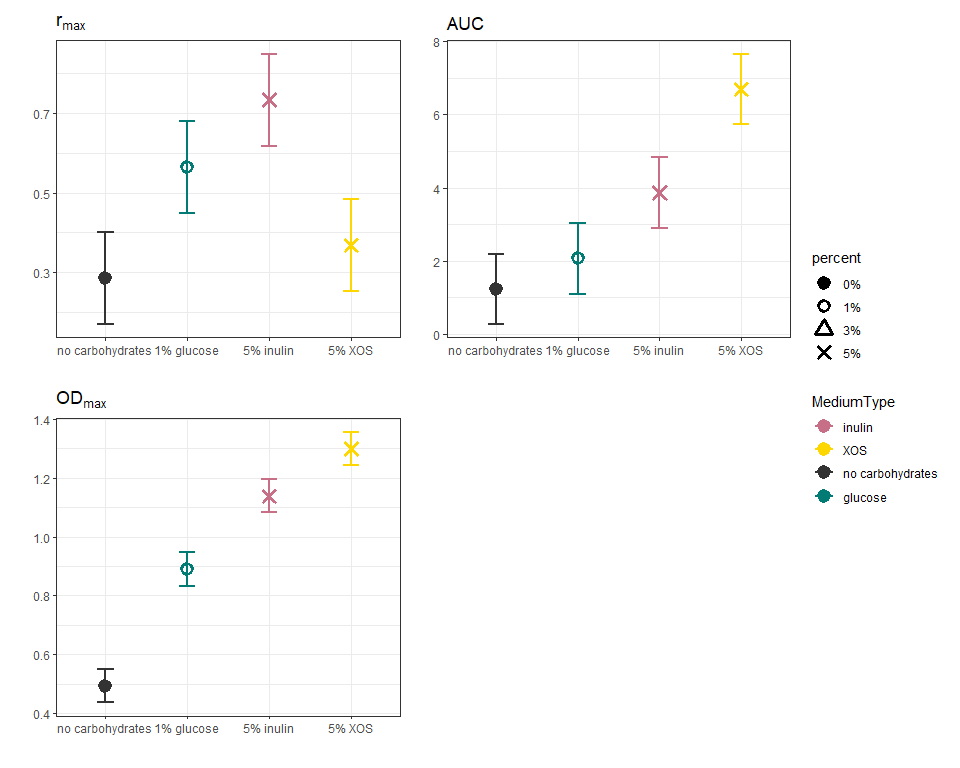

Supplement: Supplementary file 1 [file ijms-24-12796-s001.zip › supplementary-figureS2_salivarius_boxplots.png]

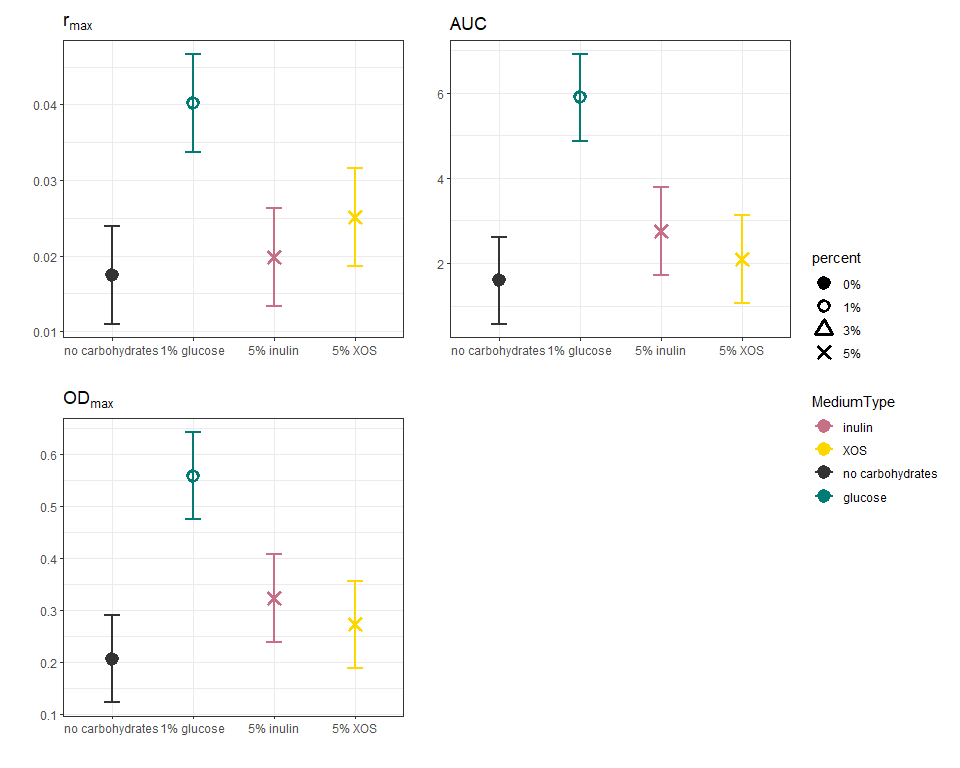

Supplement: Supplementary file 1 [file ijms-24-12796-s001.zip › supplementary-figureS2_unifomis_boxplots.png]
